# EphB4 as a therapeutic target in mesothelioma

**DOI:** 10.1186/1471-2407-13-269

**Published:** 2013-05-30

**Authors:** Ren Liu, Benjamin D Ferguson, Yue Zhou, Kranthi Naga, Ravi Salgia, Parkash S Gill, Valery Krasnoperov

**Affiliations:** 1 School of Medicine, University of Southern California, 1441 Eastlake Avenue, Los Angeles, CA 90033, USA; 2 Pritzker School of Medicine, University of Chicago, 5841 S. Maryland Avenue, Chicago, IL 60637, USA; 3 VasGene Therapeutics Inc, 1929 Zonal Avenue, Los Angeles, CA 90033, USA

**Keywords:** EphB4, Mesothelioma, sEphB4, Cancer therapy

## Abstract

**Background:**

Malignant pleural mesothelioma (MPM) often develops decades following exposure to asbestos. Current best therapy produces a response in only half of patients, and the median survival with this therapy remains under a year. A search for novel targets and therapeutics is underway, and recently identified targets include VEGF, Notch, and EphB4-Ephrin-B2. Each of these targets has dual activity, promoting tumor cell growth as well as tumor angiogenesis.

**Methods:**

We investigated EphB4 expression in 39 human mesothelioma tissues by immunohistochemistry. Xenograft tumors established with human mesothelioma cells were treated with an EphB4 inhibitor (monomeric soluble EphB4 fused to human serum albumin, or sEphB4-HSA). The combinatorial effect of sEphB4-HSA and biologic agent was also studied.

**Results:**

EphB4 was overexpressed in 72% of mesothelioma tissues evaluated, with 85% of epithelioid and 38% of sarcomatoid subtypes demonstrating overexpression. The EphB4 inhibitor sEphB4-HSA was highly active as a single agent to inhibit tumor growth, accompanied by tumor cell apoptosis and inhibition of PI3K and Src signaling. Combination of sEphB4-HSA and the anti-VEGF antibody (Bevacizumab) was superior to each agent alone and led to complete tumor regression.

**Conclusion:**

EphB4 is a potential therapeutic target in mesothelioma. Clinical investigation of sEphB4-HSA as a single agent and in combination with VEGF inhibitors is warranted.

## Background

Malignant pleural mesothelioma (MPM) is a uniformly fatal disease. It originates from normal mesothelial cells lining the pleural or peritoneal cavity long after exposure to asbestos [1,2]. There are three main histological types of malignant mesothelioma (epitheloid, sarcomatoid, and mixed or biphasic), with longer survival in epitheloid and shorter survival in sarcomatoid types [3]. Nearly 3,000 new cases are diagnosed each year in the United States [2]. The median overall survival of MPM patients ranges from 12 to 24 months [3].

The most effective treatment regimen (cisplatin and pemetrexed) induces partial response in half of patients and improves survival from 9 to 12 months [4]. Novel targeted therapies have been investigated in MPM with limited success, including vascular endothelial growth factor (VEGF) inhibitors [5,6]. Discovery of additional targets and rational combinations of targeted therapies may lead to effective novel therapies. The type 1 receptor tyrosine kinase EphB4 and its cognate ligand Ephrin-B2 are a pair of potential novel targets.

EphB4 and Ephrin-B2 are normally expressed on endothelial cells of venous and arterial lineage, respectively, and their interaction is critically required for new vessel formation, fusion between vessel compartments, and blood flow [7,8]. In addition, Ephrin-B2 is also expressed on pericytes and vascular smooth muscle cells, where it plays critical role in vessel maturation [9,10]. In tumor angiogenesis, loss of Ephrin-B2 leads both to significantly reduced tumor vessel density and to tumor growth [11-14]. EphB4, on the other hand, is overexpressed in a variety of epithelial cancers, including breast, prostate, ovarian, esophageal, colon, and head and neck cancers [15-23]. Importantly, we have also shown that EphB4 is expressed in mesothelioma and provides a survival advantage to tumor cells [24].

Both EphB4 and Ephrin-B2 are transmembrane proteins and direct cell-cell contact leads to bidirectional signaling. EphB4 activation leads to downstream activation of the phosphoinositide kinase-3 (PI3K) pathway in tumor cells [20], while Ephrin-B2 activation leads to activation of Src [25,26]. Inhibition of EphB4-Ephrin-B2 signaling blocks tumor angiogenesis, which in turn leads to hypoxia and induces VEGF expression [14]. Targeting VEGF and EphB4-Ephrin-B2 simultaneously is thus a potentially effective therapy.

In this study, we investigated the aberrant expression of EphB4 in a cohort of primary MPM tissues. We show that a significant proportion of MPM tumors expressed EphB4, which provides survival advantage to tumor cells. We also investigated the efficacy of sEphB4-HSA as an inhibitor of EphB4-Ephrin-B2 in MPM xenograft models. sEphB4-HSA induces cell death in MPM tumor xenografts *in vivo* and down-regulates major signaling pathways including PI3K and Src. In addition, we demonstrate that the combination of sEphB4-HSA and VEGF antibody has superior efficacy than either single agent alone, leading to complete tumor regression. Based on these promising preclinical results, future clinical investigation of the efficacy of sEphB4-HSA combined with VEGF inhibitors in MPM is warranted.

## Methods

### Materials

Soluble EphB4 cDNA fused in-frame with human serum albumin cDNA [14] was expressed as a seamless fusion protein in CHO cells and purified to homogeneity. EphB4-specific antibody (MAb131) was produced by VasGene Therapeutics Inc. Bevacizumab (Genentech Inc) was purchased. Phosphorylated AKT (Ser473), S6 (Ser235/Ser236) and Src (Tyr416) antibodies were from Cell Signaling, Ki67 antibody was from Abcam, CD31 and NG2 antibodies were from BD Biosciences, and terminal deoxynucleotidyl transferase–mediated dUTP nick end labeling (TUNEL) fluorescent kit was from Promega.

### Cell lines

NCI-H2373 and MSTO-211H mesothelioma cell lines were obtained from American Type Culture Collection (Manassas, VA). Cells were maintained in RPMI 1640 supplemented with 10% heat-inactivated fetal bovine serum (FBS; Life Technologies, Gaithersburg, MD) and penicillin/streptomycin (Invitrogen, Carlsbad, CA).

### Immunohistochemistry

Formalin-fixed paraffin-embedded malignant mesothelioma tumors were analyzed. Tissue analysis was approved by the institutional review board. 4-μm sections were deparaffinized, rehydrated, and washed with TBS/Tween-20. Antigens were retrieved with exposure to 1 mM EDTA (pH 8.0; DakoCytomation) for 20 minutes. Endogenous peroxidase activity in samples was blocked by exposure to 3% hydrogen peroxide/PBS (Fisher Scientific, Fair Lawn, NJ) and serum-free protein block (DakoCytomation). Tissue sections were incubated with primary antibodies overnight at 4°C. Standard avidin/biotin immunoperoxidase methods with diaminobenzidines as the chromogen were used for detection (DakoCytomation). The intensity of staining was quantified with ImageJ (NIH). EphB4-specific monoclonal mouse anti-human antibody MAb131 was used for MPM tissues. Positive controls included the 293T cell line stably expressing full-length EphB4. Negative controls included co-incubation of tissues with primary antibody and immunizing peptide.

### In vivo *tumor growth studies*

Male BALB/c *nu/nu* mice (9 weeks old) were injected with 5 × 10^6^ tumor cells in the flank. When tumor sizes reached 150 mm^3^, mice were grouped (8 tumors per group) and treated with intraperitoneal (i.p.) injection of PBS (control, 3 times per week), sEphB4-HSA (20 mg/kg, 3 times a week), Bevacizumab (20 mg/kg, 3 times a week), or a combination of sEphB4-HSA and Bevacizumab. Tumor volume was measured three times a week and calculated using the following formula: tumor volume = 0.52 × length × width^2^, where length and width are the longest and shortest dimensions of a palpable tumor. All procedures were approved by Institutional Animal Care and Use Committee and performed in accordance with the Animal Welfare Act regulations.

### Immunofluorescence

Xenograft tumors were harvested and immediately snap frozen. 5-μm fresh frozen tissue sections were fixed in phosphate-buffered 4% paraformaldehyde, blocked with goat serum, and incubated with primary antibody overnight at 4°C. Antibody binding was localized with appropriate AlexaFluor-conjugated secondary antibodies (Invitrogen, Carlsbad, CA). Nuclei were counterstained with 6-diamidino-2-phenylindole dihydrochloride hydrate (DAPI). Images were obtained with a Nikon Eclipse 80i fluorescence microscope and Meta Morph imaging series system. The intensity of staining and the positive signal coverage area were quantified with ImageJ (NIH).

### Statistics

A student’s *t*-test (two-tailed, unpaired) was used to calculate P values between groups where indicated.

## Results and discussion

### EphB4 overexpression in MPM tumors

**Figure 1:**
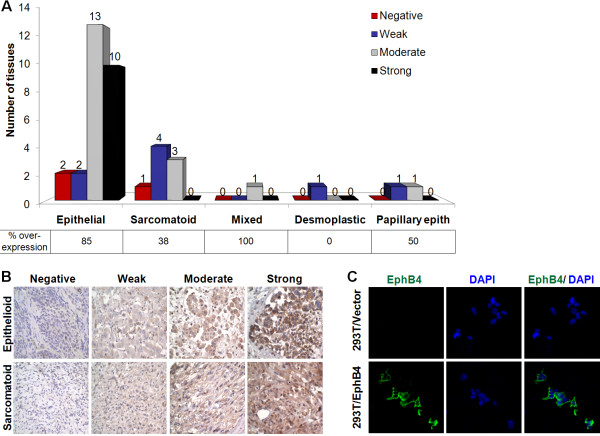
**EphB4 overoverexpression in MPM.** (**A**) A panel of MPM tissues was stained with EphB4-specific antibody MAb131, and EphB4 expression level was scored and summarized. Moderate and strong expression was considered overexpression. (**B**) Representative images demonstrating EphB4 expression patterns in epithelioid and sarcomatoid subtypes. (**C**) 293T cells grown on 8-well chamber slide were transfected with human EphB4 overexpresion vector or empty vector pCDNA3.1. 2 days after transfection, cells were fixed with 4% paraformaldehyde and stained with MAb131 that was also used for staining in (**A**). Nuclei were counter-stained with DAPI.

39 human MPM tumor samples consisting of 27 epithelioid, 8 sarcomatoid, 2 papillary epithelioid, 1 mixed, and 1 desmoplastic subtypes were studied for EphB4 expression by immunohistochemistry. 85% of epithelioid, 38% of sarcomatoid, and 100% of mixed cell type were positive for EphB4 overexpression (moderate or strong staining, Figure 1A). The representative IHC pictures of EphB4 expression in epithelioid and sarcomatoid MPM subtypes are shown in Figure 1B. In light of personalized therapeutics, this observation that EphB4 overexpression is more frequent in epithelioid tissues is important for clinical study design with therapeutic compounds targeting EphB4 and consideration to select appropriate cases.

The EphB4 antibody (MAb131) used for mesothelioma tissue staining has been shown to have no cross-reactivity to other EphB receptors [27]. To further confirm its specificity in staining, we used it to stain 293T cells ectopically expressing EphB4 and 293T cells transfected with empty vector. Only EphB4 overexpressing 293T cells showed signal (Figure 1C), confirming the specificity of MAb131.

### In vivo *activity of sEphB4-HSA in a xenograft model of malignant mesothelioma*

sEphB4 is a soluble decoy of EphB4 that blocks EphB4-Ephrin-B2 bi-directional signaling [28]. sEphB4-HSA has full-length human serum albumin fused to the C-terminus of sEphB4 to improve half life and delivery [14,29]. We have previously shown that both variants of the protein the sEphB4 alone and sEphB4-HSA have anti-tumor activities in multiple tumor models [14,28,30].

**Figure 2:**
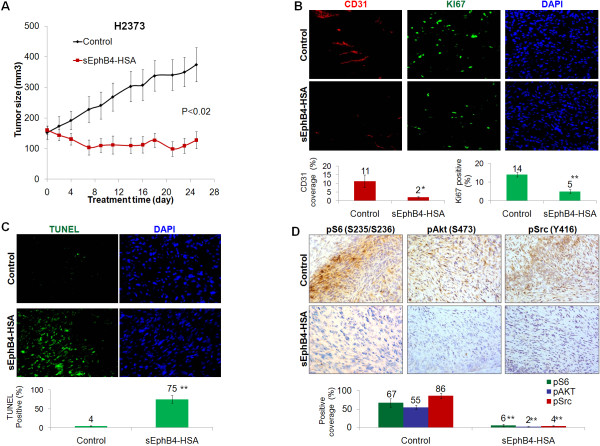
**sEphB4-HSA inhibited proliferation and induced apoptosis of MPM cell *in vivo*.** (**A**) sEphB4-HSA (20 mg/kg, 3 times a week) profoundly inhibited H2373 tumor growth *in vivo*. (**B**) Representative images of CD31 and Ki67 staining of harvested H2373 tumors showing reduced vessel density and tumor cell proliferation by sEphB4-HSA treatment. CD31 and Ki67 coverage were normalized to DAPI coverage. (**C**) Representative images of TUNEL staining of harvested H2373 tumors showing induced tumor cell apoptosis by sEphB4-HSA treatment. TUNEL coverage was normalized to DAPI coverage. (**D**) Representative images of phosphorylated Akt, S6, and Src staining of harvested H2373 tumors showing inhibited PI3K and Src signaling by sEphB4-HSA treatment. At least 4 images from each analysis were used for quantification and statistical analysis. *, P<0.01; **, P<0.002. Error bars indicate standard error of mean.

Utilizing anti-sense oligonucleotides against EphB4, we had previously reported decreased survival of mesothelioma cell lines [24]. Here, we studied the antitumor activity of sEphB4-HSA in mesothelioma xenograft models using the human sarcomatoid mesothelioma cell line H2373 that has robust overexpression of EphB4. Sarcomatoid is also a category of mesothelioma that is the most difficult to treat. After 25 days of treatment, mice receiving sEphB4-HSA had a 66% reduction in tumor volume (Figure 2A, *P* < 0.02) compared to control group, and a 20% regression from the starting tumor volume (P < 0.05). Tissues harvested at the conclusion of the study showed reduced vessel density (18% of control; Figure 2B), cell proliferative index (36% of control; Figure 2B), and increased apoptosis (19-fold increase over control; Figure 2C). Thus, blockade of EphB4 activation inhibited tumor angiogenesis and mesothelioma cell proliferation and also induced mesothelioma cell apoptosis *in vivo*.

The PI3K pathway is a major pathway downstream of EphB4 and a critical pathway in mesothelioma [20,31]. It is also known that activation of the PI3K pathway increases EphB4 levels [15], thus establishing a positive feedback loop. Here, sEphB4-HSA-treated MPM tumors showed marked decreases in PI3K signaling measured by the downstream levels of phophorylated Akt (Ser473) and phosphorylated ribosomal protein S6 (Ser235/Ser236; Figure 2D). sEphB4-HSA also reduced phosphorylation of Src, which signals downstream of Ephrin-B2 activation [25,26]. Activated Src induces Bcl-xL and Mcl-1 [32]; thus, inhibition of Src activation may contribute to the sEphB4-HSA-induced apoptosis seen here. In addition, inhibition of phosphorylation of Akt, S6, and Src was observed at the end of the 25-day treatment, suggesting that the treatment did not trigger a rebound effect.

### Combination of sEphB4-HSA and VEGF antibody

**Figure 3:**
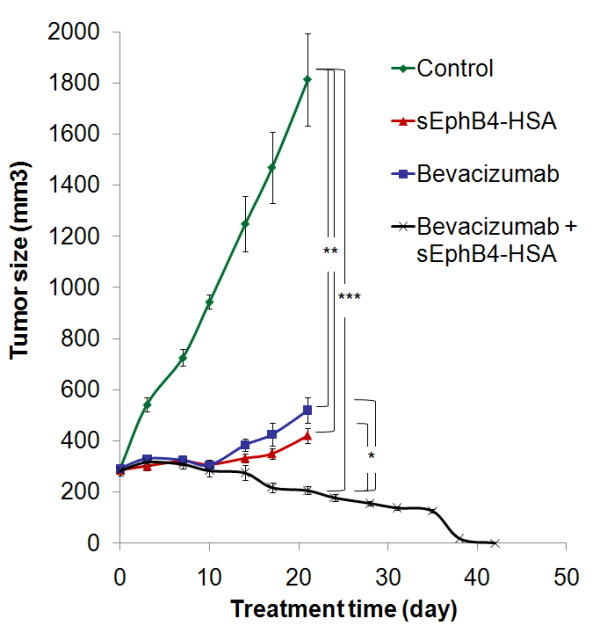
***In vivo *efficacy of sEphB4-HSA combined with Bevacizumab.** 211H tumors were treated with sEphB4-HSA alone (20 mg/kg, 3 times a week), Bevacizumab alone (20 mg/kg, 3 times a week), or sEphB4-HSA combined with Bevacizumab. PBS was used as control. Treatment in single-agent groups and control group was continued for 21 days, whereas treatment in the combination group was continued for 42 days until complete tumor regression. *, P<0.05; **, P<0.02; ***, P<0.01. Error bars indicate standard error of mean.

We have shown previously that VEGF and VEGFRs are expressed in MPM, which is one of the few tumors that utilizes VEGF as an autocrine growth factor [33]. sEphB4-HSA has been shown to markedly inhibit tumor angiogenesis leading to elevated tumor VEGF levels [14], suggesting that the combination of sEphB4-HSA with VEGF inhibition may lead to enhanced anti-angiogenesis and antitumor activity. We thus combined sEphB4-HSA and the VEGF-neutralizing antibody Bevacizumab in a xenograft study. In light of the H2373 data, we tested another mesothelioma cell line, 211H. This cell line also overexpresses EphB4 and was derived from a patient with biphasic histology, a hard to treat category of mesothelioma [24]. Both agents were highly active alone (71% and 77% reduction in tumor volume at day 21 compared to control group for Bevacizumab and sEphB4-HSA, respectively), while combination therapy led to complete regression of the established tumor after 38 days of treatment (Figure 3), suggesting that this combination warrants further clinical investigation as a therapeutic regimen.

**Figure 4:**
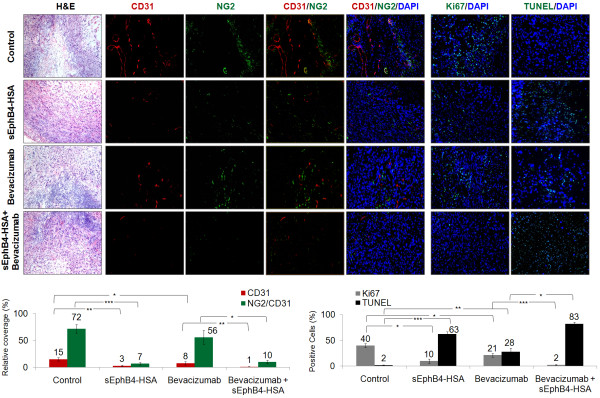
**Combinatorial effects of sEphB4-HSA and Bevacizumab on tumor vasculature and tumor cells.** 211H tumors were treated as in Figure 3 for one week. Tumors were then harvested for immunofluorescence analysis. Tumor vessel density was evaluated by CD31 staining. Pericyte recruitment to tumor vessel was evaluated by colocalization of NG2-positive pericytes and CD31-positive endothelial cells. Cell proliferative index was evaluated by Ki67 staining and apoptosis was assessed with TUNEL staining. At least 4 images from each analysis were used for quantification and statistical analysis. *, P<0.05; **, P<0.01; ***, P<0.002. Error bars indicate standard error of mean.

Since tumors went to complete remission after combinatorial treatment of sEphB4-HSA and Bevacizumab, we had no tumor tissues for analysis. Therefore, we performed a one-week treatment of 211H tumors to analyze the mechanism of the combinatorial effects of sEphB4-HSA and Bevacizumab (Figure 4). After one week treatment, sEphB4-HSA and Bevacizumab each alone reduced vessel density (P < 0.01 and P < 0.05, respectively). sEphB4-HSA had much greater inhibition of tumor vessel density than Bevacizumab, which is consistent with our previous study in Kaposi’s Sarcoma [14]. However, tumor growth inhibitions after 3 weeks of treatment with sEphB4-HSA and Bevacizumab were similar (Figure 3). This is very likely due to Bevacizumab’s effects on tumor cells directly - mesothelioma is one of only few tumor types that express VEGFR2, produce VEGF and thus have an autocrine loop [33].

We also observed that vessels in Bevacizumab-treated tumors appeared normal in caliber while sEphB4-HSA led to not only significantly reduced vessel density, but also markedly reduced vessel length, vessel caliber, and pericyte recruitment (measured by co-localization of NG2-positive pericytes and CD31-positive endothelial cell; P < 0.002) compared to Bevacizumab treatment.

Ephrin-B2 is strongly expressed on tumor vessels and is necessary for maintaining tumor vasculature. The EphB4-Ephrin-B2 inhibitor sEphB4-HSA prevents EphB4-induced Ephrin-B2 activation in MPM tumor cells. This blockade has two outcomes on tumor vasculature. First, since Ephrin-B2 binds directly to VEGFRs and its activation is required for VEGFR activation [11,13], EphB4-Ephrin-B2 blockade will inhibit VEGF-induced signaling. Second, pericyte recruitment and vessel maturation will be impaired because Ephrin-B2 is also expressed on pericytes and is necessary for these processes. sEphB4-HSA treatment thus leads to not only fewer but also less mature tumor vessels. When sEphB4-HSA and Bevacizumab were combined, vessel density is further decreased (P < 0.01 compared to Bevacizumab alone), along with diminished pericyte recruitment (P < 0.05 compared to Bevacizumab alone). This enhanced anti-angiogenesis activity combined with direct antitumor activity led to more significantly reduced proliferation (Ki67 staining, P < 0.002 compared to Bevacizumab alone) and increased apoptosis (TUNEL staining, P < 0.05 compared to Bevacizumab alone) than either single agent alone (Figure 4). This may explain the complete regression seen in the long-term combinatorial treatment.

## Conclusions

In this study, we found that EphB4 is highly expressed in MPM, especially in epithelioid subtype, and represents a potential therapeutic target. We also found that the EphB4-Ephrin-B2 inhibitor sEphB4-HSA, alone or combined with the anti-VEGF antibody Bevacizumab was highly active in inhibiting mesothelioma growth in xenograft models. sEphB4-HSA is currently in a clinical Phase 1 trial (ClinicalTrials.gov Identifier: NCT01642342). The data presented here suggest that mesothelioma should be a target disease in clinical investigation of this novel therapy, and the combination of sEphB4-HSA and Bevacizumab should undergo further clinical investigation.

## Competing interests

Kranthi Naga and Valery Krasnoperov are employees of VasGene Therapeutics Inc.

## Authors’ contributions

RL and YZ carried out the xenograft studies and immunoanalysis of tumor tissues. BF performed immunoanalysis of human MPM tissues. KN and VK produced EphB4 antibody and sEphB4-HSA for this study. RS, PSG, and VK participated in the design of the study. RL, BF, RS, PSG, and VK drafted the manuscript. All authors read and approved the final manuscript.

## Pre-publication history

The pre-publication history for this paper can be accessed here:

http://www.biomedcentral.com/1471-2407/13/269/prepub
